# The Influence of Shuttle-Shape Emodin Nanoparticles on the *Streptococcus suis* Biofilm

**DOI:** 10.3389/fphar.2018.00227

**Published:** 2018-03-13

**Authors:** Wenya Ding, Jin Sun, He Lian, Changgeng Xu, Xin Liu, Sidi Zheng, Dong Zhang, Xiaopeng Han, Yanyan Liu, Xueying Chen, Bello O. God′spower, Yanhua Li

**Affiliations:** ^1^Department of Veterinary Medicine, Northeast Agricultural University, Harbin, China; ^2^Heilongjiang Key Laboratory for Animal Disease Control and Pharmaceutical Development, Harbin, China; ^3^School of Pharmacy, Shenyang Pharmaceutical University, Shenyang, China

**Keywords:** emodin nanoparticles, *Streptococcus suis* biofilm, biofilm inhibition, biofilm elimination, drug uptake

## Abstract

Biofilm is one of the most important physiological protective barriers of the *Streptococcus suis* (*S. suis*), and it is also one of the primary causes of hindrance to drug infiltration, reduction of bactericidal effects, and the development of antibiotic resistance. In order to intervene or eliminate *S. suis* biofilm, shuttle-shape emodin-loaded nanoparticles were developed in our study. The emodin nanoparticles were prepared by emodin and gelatin–cyclodextrin which was synthesized as drug carrier, and the nanoparticles were 174 nm in size, -4.64 mv in zeta potential, and exhibited a sustained emodin release. Moreover, the delivery kinetics of nanoparticles were also explored in our study. The confocal laser scanning microscopy and colony forming unit enumeration experiment indicated that nanoparticles could increase drug infiltration and uptake by biofilm. The flow cytometry system analysis showed that nanoparticles could be up taken by 99% of the bacteria cells. TCP assay and scanning electron microscopy showed that the nanoparticles had better effect on biofilm inhibition and elimination when compared with emodin solution. These results revealed that the emodin nanoparticles had a better therapeutic effect on the *S. suis* biofilm *in vitro*.

## Introduction

*Streptococcus suis* is an important zoonotic pathogen causing arthritis, meningitis, bronchopneumonia, septicemia, and even sudden death in pigs and humans (Tang et al., 2006). Human *S. suis* infection can be acquired after any contact with contaminated pigs or pig-derived products (Schultsz et al., 2012). And the diseases caused by *S. suis* are difficult to cure, especially the *S. suis* serotype 2 which can form biofilm (Wang et al., 2011a). It is because biofilm can render their inhabitants more resistant to disinfectant and cause persistent infection (Chajecka-Wierzchowska et al., 2016).

The biofilm is a bacterial community structure and the cells are enclosed by self-produced polymeric matrix which includes exopolysaccharides, proteins, nucleic acids, and other substances (Anwar et al., 1990; Costerton et al., 1999; Matz et al., 2004). These matrix components play a key role in increasing bacteria’s resistance to drug, because they serve as a protective membrane to reduce the drug uptake and retard drug diffusion in biofilm. Antibacterial resistance to biofilm-growing bacteria can be up to a 1000-fold in magnitude when compared with their planktonic counterparts (Cai et al., 2015). So biofilm is one of the major causes of poor healing in *S. suis* infection.

Emodin (1,2,8-trihydroxy-6-methylanthraquinone), a natural compound extracted from rhubarb, has many biological activities, such as anti-inflammatory (Han et al., 2015), antibacterial (Liu et al., 2013), immunosuppressive, and inhibition of biofilm (Han et al., 2015). It can hydrolyze quorum-sensing signal receptor TraR, and significantly inhibit biofilm formation in *Escherichia coli* (Ding et al., 2011). The emodin can significantly downregulate *luxS* gene (Yang et al., 2015), and the *luxS* gene is involved in the production of autoinducer-2, a signal molecule playing a role in biofilm formation (Kuehl et al., 2009). It can also reduce *S. mutans* biofilm formation on hydroxyapatite by insertion of the planar molecule into the cell membrane and/or by binding of the same molecule to membrane-embedded molecules (Coenye et al., 2007). Our preliminary study revealed that the biofilm of *S. suis* was significantly inhibited by sub-MIC of emodin and the expression of virulence factors in *S. suis* was also affected by emodin (Yang et al., 2015).

In this study, nanoparticle-forming biomaterial (Gel-CD) was synthesized by conjugating the gelatin with the cyclodextrin (CD), and emodin-loaded nanoparticles were also prepared. The emodin nanoparticles exhibited many attractive features, such as small particle size, high drug encapsulation efficiency (EE), drug-loading (DL) with amorphous state, and sustained release of characteristics. Most importantly, emodin nanoparticles exhibited higher inhibition to biofilm formation and effect on biofilm elimination. And we also explored the reason for the improvement of the effect on biofilm elimination by emodin nanoparticles.

## Materials and Methods

### Materials

Gelatin A and CD were purchased from Sigma-Aldrich Co. (St. Louis, MO, United States) and Sinopharm Chemical Reagent Co., Ltd. (Shenyang, China) respectively. *N*-(3-dimethylaminopropyl)-*N*′-ethyl-carbodiimide hydrochloride (EDC) was obtained from Chengdu Luhkesen Co., Ltd. (Chengdu, China). Acetone, dimethylformamide (DMF), and *p*-toluene sulfonylchloride were purchased from Yuwang Co., Ltd. (Shandong, China). Todd–Hewitt broth (THB) was purchased from Sigma-Aldrich Co. (St. Louis, MO, United States). Emodin was purchased from Chengdu Herbpurify Co., Ltd. (Chengdu, China). All other chemicals and reagents were of analytical grade and used without further purification.

### Synthesis of Gel-CD

#### Synthesis of Tosylatation Cyclodextrin

*p*-Toluene sulfonylchloride 7.5 g was dissolved in DMF and CD 5.0 g was added to the solution. The mixture was constantly stirred at 45°C for 24 h. To obtain tosylatation CD, acetone was added and the sample was deposited. The precipitate was rinsed twice with acetone and the tosylatation CD was obtained as white powder.

#### Synthesis of Gel-CD

To synthesize the Gel-CD, gelatin A 0.5 g was initially activated by 0.2 g EDC in DMF and the tosylatation CD 5 g was slowly added, then the mixture was stirred at 45°C for 2 days. The resulting solution was dialyzed against the excess amount of DMF, CD, and EDC for 7 days with deionized water. After being freeze-dried, the Gel-CD copolymer was isolated as a canary yellow floccule.

### Characterization of Gel-CD Copolymer

#### NMR Spectroscopy of Gel-CD Copolymer

The structure of Gel-CD copolymer was analyzed by ^1^H-NMR and the spectrum was recorded on a Bruker spectrometer operated at a frequency of 300 MHz for protons with D_2_O as the solvent.

#### CD Content of the Gel-CD Polymer

The CD content in the Gel-CD copolymer was determined by UV–vis spectrophotometry. Firstly, 10 mg Gel-CD copolymer was dissolved in 10 mL PBS (pH 10.5) and 0.1 mg phenolphthalein was added, then the mixture was incubated at room temperature for 30 min, and the absorbance was measured at a wavelength of 553 nm with UV–vis spectrophotometer (UV-1800, Ruili, China). Finally, working standard solutions of CD were prepared with concentration range from 19.72 to 197.20 μg/mL and tested as described above, then the calibration curve was calculated.

#### Determination of the Critical Micelle Concentration (CMC) of Gel-CD Copolymer

The CMC of Gel-CD copolymer can be tested by fluorescence spectroscopy using pyrene as a fluorescent probe. Pyrene was dissolved in ethanol, then the mixture was added to test tubes and the ethanol was evaporated by nitrogen. Then, Gel-CD copolymer solutions with a series of concentrations which ranged from 10^-1^ to 10^-6^ mg/ml were added into the test tubes to attain a final pyrene concentration of 3 × 10^-7^ mol/L. The mixtures were sonicated for 4 h and incubated at room temperature for 12 h. Then the solutions were measured by microplate reader (BIO-RAD 680, United States; excitation wavelength: 333 and 335 nm and emission wavelength: 339 nm). The intensity ratio of different wavelength (*I*_333_/*I*_335_) was plotted as a function of the Gel-CD copolymer concentrations and the CMC was inferred from the cross-point.

### Preparation of Emodin Nanoparticles

Emodin nanoparticles were prepared by the sonicate method. Twenty milligrams of Gel-CD was dissolved in 5 mL water and 2 mL emodin solution (1 mg/mL in methanol) was slowly added. After stirring for about 30 min at room temperature, the methanol was removed by rotary vacuum evaporation. Then the mixture was sonicated by a probe-type sonifier (JY92-2D, Scientz, China) for 10 min in an ice bath to keep it cool. The solution was centrifuged at 4,000 rpm for 10 min and then passed through 0.45 mm filters to remove the untrapped drug, then the final product was obtained.

### Characterization of Emodin Nanoparticles

#### Size and Zeta Potential

The particle size, particle size distribution, and zeta potential of emodin nanoparticles were tested by Zetasizer (NanoZS, Malvern Co., United Kingdom). The emodin nanoparticles were analyzed in 1 mL distilled water at 25°C and the measurements were repeated in triplicate.

#### Emodin Nanoparticles Morphology

The morphology of the emodin nanoparticles was observed by transmission electron microscopy (TEM) (H-600, Hitachi, Tokyo, Japan). A drop of emodin nanoparticles solution was dropped into carbon-coated copper grid and the excess solution was removed with a filter paper, and then the nanoparticles were investigated by TEM.

#### Drug-Loading (DL) Content and Encapsulation Efficiency (EE)

The DL and EE of emodin nanoparticles were measured by a UV–vis spectrometer. Emodin nanoparticles solution was diluted 10-fold with 50% ethanol and sonicated to disassemble nanoparticles, the solution was detected by UV–vis spectrophotometer (UV-1800, Ruili, China) at 438 nm.

### *In Vitro* Release of Emodin From Emodin Nanoparticles

The drug release from nanoparticles was measured by dialysis bag diffusion method. One milliliter of emodin nanoparticles suspension in water with emodin concentration of 0.4 mg/mL was placed into a pretreated dialysis bag (MWCO: 8–10 kDa) and immersed in 200 mL phosphate buffer saline (pH 7.4, containing 30% ethanol). The release was conducted in an incubator shaker set at 100 rpm/min and 37°C. At predetermined time points, 100 μL release medium was withdrawn for examining and replaced by equivalent volume of fresh medium in the flask. The drug was determined by a valid High-Performance Liquid Chromatography (HPLC) method: which comprised of a UV–vis L-7420 Detector, L7110 Pump, L-7200 Auto sampler, and a wavelength of 254 nm. The stationary phase was composed of C_18_ column (200 mm × 4.6 mm, 5 μm; Dikma Technologies, China) reversed phase, the mobile phase included methanol and phosphoric acid (85:15, v/v), the flow rate was 1.0 mL/min with a column temperature of 25°C, and an injection volume of 20 μL. The accumulative weight and relative percentage of the released emodin were calculated as the function of incubation time. And the released amount was analyzed using first-order kinetic model, Higuchi model, and Ritger–Peppas model. The equation of first-order kinetic model: *Q* = *Q*_0_(1-e*^kt^*), Higuchi model: *Q* = *kt*^1/2^, Peppas equation: *Q* = *kt^n^, Q* is the drug fraction released at time *t, Q*_0_ is the content of medicine, *k* is a constant reflecting the structural and geometric characteristics of the nanoparticles, and *n* is the release exponent, which indicates the drug release mechanism.

### Bacterial Culture and Biofilm Formation

#### Bacterial Culture and Biofilm Formation

*Streptococcus suis* strain ATCC 700794 was grown overnight in THB (Todd–Hewitt yeast Broth) (Sigma-Aldrich, Co., St. Louis, MO, United States) containing (w/v) 0.5% beef extract, 2% peptone, 0.3% yeast extract, 2% calf serum, 0.2% NaCl, 0.04% Na_2_HPO_4_, 0.25% Na_2_CO_3_, and 0.5% glucose (Zhao et al., 2015). Overnight bacterial culture was diluted to an optical density of 0.2 at 600 nm (OD_600_) by THB (1 × 10^6^ CFU/mL). Then, 200 mL aliquots were added into the wells of a sterile 96-well polystyrene microtitre plate and incubated at 37°C for 72 h.

#### Scanning Electron Microscopy (SEM)

The *S. suis* ATCC 700794 biofilm was tested by SEM as described by Zhao et al. (2015). A mid-exponential growth culture of *S. suis* was diluted to an optical density of 0.2 at 600 nm (OD_600_) and 2 mL was added to a 6-well microplate (CBD) wells containing a 11 mm × 11 mm sterilized rough organic membrane (Mosutech Co., Ltd., Shanghai, China) at the bottom. After incubation at 37°C for 72 h without shaking, the supernatant was removed and the organic membranes were rinsed with sterile PBS. The biofilm was fixed with 4% glutaraldehyde for 6 h and washed three times with 0.1 M PBS, then fixed in 2% osmium tetroxide. After dehydrating and critical point drying, samples were sputtered gold with ion sputtering instrument (current 15 mA, 2 min) and examined by SEM (FEI Quanta, Netherlands).

### Effect of Emodin Nanoparticles on Biofilm Formation

Minimum inhibitory concentrations (MIC) of *S. suis* were separately determined for the emodin, emodin nanoparticles, and Gel-CD copolymer by the microtitre method as described in the Clinical and Laboratory Standards Institute (CLSI) guidelines but replacing Mueller–Hinton broth by THB (Król et al., 2016). The emodin or emodin nanoparticles concentrations were 50, 25, 12.5, 6.25, 3.12, 1.56, 0.78, and 0.39 μg/mL, and the Gel-CD copolymer were 4, 2, 1, 0.5, 0.25, 0.12, 0.06, and 0.03 mg/mL. In addition, a negative control (with bacteria alone) and a positive control (with THB alone) were also included.

One hundred microliters of the *S. suis* suspension and 100 μL of drugs were added to each well of a 96-well microplate and the final concentrations of each emodin nanoparticles and emodin were 1/2× MIC (1.56 and 3.12 μg/mL), 1/4× MIC (0.78 and 1.56 μg/mL), 1/8× MIC (0.39 and 0.78 μg/mL), and 1/16× MIC (0.19 and 0.39 μg/mL), respectively. In addition, a negative control (with bacteria alone) was also included. After incubation for 72 h without shaking, biofilm was measured by the TCP assay (Wang et al., 2011b)^.^ The supernatants in the 96-well microplate were removed, and the wells were rinsed with 50 mM PBS (pH 7.2) and fixed by 200 μL methanol for 30 min, then stained with 200 μL 1% crystal violet (w/v) for 30 min, the wells were rinsed three times with PBS (pH 7.2) and dried for 2 h at 37°C. Then, 200 μL 33% acetic acid (v/v) was added and the plate was shaken for 10 min. All the wells were measured by Tecan GENios Plus Microplate Reader (Tecan, Austria) at 595 nm.

Sub-MIC emodin and emodin nanoparticles were added into the *S. suis* suspension after incubating 0, 24, and 48 h, respectively. In addition, a negative control (with bacteria alone) was also included. After incubation for 72 h without shaking, the wells were tested by the TCP assay too. All assays were performed in triplicate.

The *S. sui*s and 1/2× MIC (1.56 μg/mL) emodin nanoparticles were added to a 6-well microplate (CBD) which contain a sterilized rough organic membrane at the bottom. After incubation for 72 h, the biofilm was tested by the SEM.

### Effect of Emodin Nanoparticles on Biofilm

After the *S. suis* biofilm formation, the supernatants in the 96-well microplate were removed, then emodin nanoparticles and emodin with 2× MIC (6.24 and 12.48 μg/mL), 4× MIC (12.48 and 24.96 μg/mL), and 8× MICs (24.96 and 49.92 μg/mL) were added, respectively. At the same time, a negative control (with bacteria alone) was also included. The wells were cultivated 12 h without shaking and tested by the TCP assay.

The *S. suis* biofilm was added to 8× MIC (49.92 μg/mL) emodin or 8× MIC (24.96 μg/mL) emodin nanoparticles and cultured for 3, 6, and 12 h, respectively. In addition, a negative control (with bacteria alone) was also included. The wells were tested by the TCP assay.

After the *S. suis* biofilm formation in the 6-well microplate (CBD) which contained a sterilized rough organic membrane at the bottom, 8× MIC emodin nanoparticles were added and the wells were incubated for 12 h at 37°C, then the biofilm was tested by the SEM.

### The *S. suis* Biofilm or *S. suis* Uptake of Coumarin-6 Labeled Nanoparticles

The *S. suis* biofilm in the 6-well microplate was seeded on square glass coverslips (20 mm × 20 mm), and the mature bioflim on the coverslips was obtained after incubation for 72 h at 37°C. Then, 25 μg/mL coumarin-6 solution and coumarin-6-labeled nanoparticles were added, respectively, and incubated with the biofilm for 15 min, 30 min, 1 h, and 3 h without shaking. The biofilm was washed three times with PBS (pH 7.2) at 4°C and fixed with 4% paraformaldehyde solution. Finally, the coverslips were mounted on microscope slides and analyzed under a confocal laser scanning microscope (LEICA, Germany).

A mid-exponential growth culture of *S. suis* was made to 1 × 10^7^ CFU/mL in a maximum recovery diluent, and coumarin-6 solution and the coumarin-6-labeled nanoparticles with 0.5, 2, and 5 μg/mL concentrations were added separately. At the same time, a negative control (with bacteria alone) was also included. The bacteria were cultured for 1 h, the suspensions were centrifuged, rinsed three times with PBS (pH 7.2), and tested with flow cytometry system (FCS).

### The Influence of Emodin Nanoparticles on Colony Forming Unit (CFU) in Biofilm

Overnight culture of *S. suis* was diluted to an optical density of 0.2 at 600 nm. Hundred microliters of the supernatant was added to 96-well microplate, and the emodin nanoparticles and emodin of 25 μg/mL concentrations were added. In addition, a positive control (with bacteria alone) was also included. After incubation at 37°C for 72 h without shaking, the supernatants in the 96-well microplate were removed, and the wells were rinsed with 50 mM PBS (pH 7.2). Biofilm cells were removed from the wells by sonication for 5 min in 200 μL of THB. The cell suspensions underwent 10-fold dilutions in THB, and 100 μL of each dilution was spot plated onto THB soft-agar plates and incubated at 37°C for 24 h.

### Statistical Analysis

All data were presented as mean ± SD with at least three replicates. Student’s *t*-test was applied to test the significant differences and significant level was set at *p* < 0.05.

## Results and Discussion

### Synthesis and Characterization of Gel-CD Copolymer

**FIGURE 1:**
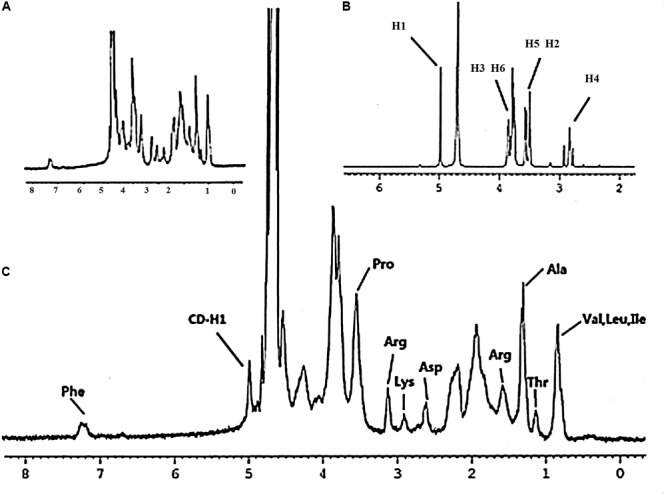
^1^H-NMR spectrum – **(A)** gelatin, **(B)** cyclodextrin, and **(C)** Gel-CD (ppm, in D_2_O).

Gel-CD copolymer was synthesized via two reaction routes. And the chemical structure of Gel-CD copolymer was confirmed by ^1^H-NMR (solvent: D_2_O). As shown in **Figure 1C**, the peak at 4.9–5.1 ppm was assigned to the protons of CD. And most of the proton signals could be assigned to the corresponding methyl resonance of amino acids from gelatin. The methyl resonance of the amino acids leucine (Leu), valine (Val), and isoleucine (Ile) was apparent at 0.87 ppm. The protons peaks at 1.17 and 1.34 ppm were assigned to the methyl resonance of threonine (Thr) and alanine (Ala). The peak at 1.61 ppm was assigned to the methyl resonance of arginine (Arg). The peaks at 2.65, 2.94, 3.16, and 3.58 ppm were derived from the amino acids aspartic acid (Asp), lysine (Lys), Arg, and proline (Pro). The resonance of the aromatic protons of tyrosine (Phe) appeared at 7.28 ppm (**Figures 1A,C**) (Bernert et al., 2011). From the NMR spectrum (**Figure 1B**), we can see that the 1H of glucose derived from CD was apparent at 4.97 ppm. The peaks at 3.750–3.852 ppm and the proton peak at 3.474–3.566 ppm were assigned to H3, H6 and H5, H2 of glucose, respectively. The peak at 2.761–2.917 ppm was ascribed to H4 of glucose. **Figure 1C** confirmed that the CD was successfully linked to gelatin. Moreover, the experiment of CD content test further confirmed this conclusion, and the regression equations of the curves and their correlation coefficients were calculated as follows: *Y* = -2.5479*X* + 0.6867 (*R*^2^ = 0.991) and the CD content of copolymer was about 15–18%.

**FIGURE 2:**
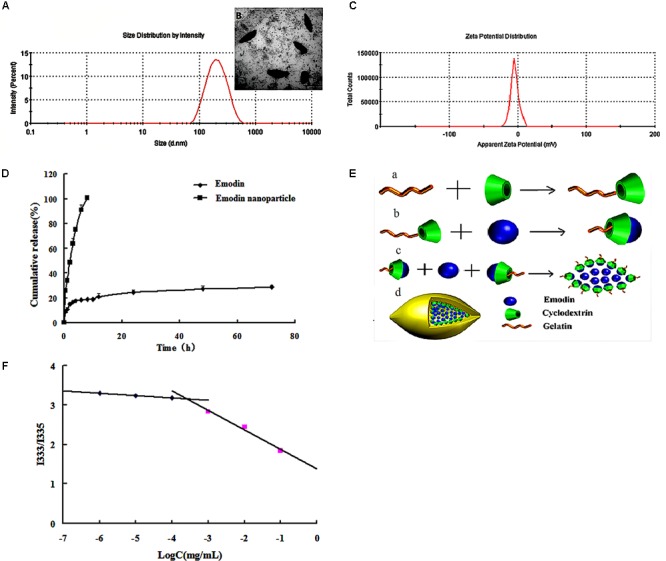
Characterization of emodin nanoparticles. **(A)** Particle size distribution of emodin nanoparticles. **(B)** TEM image of emodin nanoparticles. **(C)** Zeta potential of emodin nanoparticles. **(D)** *In vitro* release profiles of emodin and emodin nanoparticles in PBS (pH 7.4, 30% ethanol). **(E)** The formation of emodin nanoparticles. **(a)** Synthesis of Gel-CD copolymer. **(b)** The host–guest interaction between the emodin and β-CD in the Gel-CD copolymer. **(c)** The self-assembly of emodin nanoparticles. **(d)** The structure of emodin nanoparticles. **(F)** The CMC of Gel-CD copolymer.

Critical micelle concentration of Gel-CD copolymer was tested by pyrene which was used as the fluorescent probe. The curve of fluorescence intensity (*I*_333_/*I*_335_) ratio which was a function of the logarithm of Gel-CD copolymer concentration showed the CMC value of Gel-CD copolymer, the CMC was 6.5 × 10^-4^mg/mL (**Figure 2F**). It was very low, so it has an easy self-assembling process and the integrity nanoparticles under high dilution condition, such as blood circulation (Ai et al., 2014).

### Preparation and Characterization of Emodin Nanoparticles

Emodin nanoparticles were prepared by sonicate method. As illustrated in **Figure 2E**, the host–guest interaction occurred between the Gel-CD copolymer as a host molecule and the emodin as a guest molecule, then the hydrophobic core was formed by the emodin-β-CD compound and emodin, and the self-assembled emodin nanoparticles were formation.

Dynamic light scattering (DLS) and TEM images were used to test the particle size of emodin nanoparticles. **Figure 2A** showed the emodin nanoparticles with narrow distribution. The average diameter was about 174 nm and the polydispersity index was about 0.24, and the shuttle-shape emodin nanoparticles could be seen clearly in the TEM picture (**Figure 2B**). The zeta potential of emodin nanoparticles was about -4.64 mv in **Figure 2C**. The EE and DL efficiencies were 78.52 ± 0.78 and 9.06 ± 0.46%, respectively. It has been reported that emodin had anti-inflammatory (Yang et al., 2014), anti-bacterial (Cao et al., 2015), anti-virus (Xiong et al., 2011), and anti-allergic (Kim et al., 2014) effects. However, there is obvious obstacle to the development of emodin as a viable therapeutic dosage form owing to its low aqueous solubility (Shi et al., 2015). In this study, emodin was encapsulated by Gel-CD copolymer, and its aqueous solubility was significantly improved in the emodin nanoparticles.

The *in vitro* cumulative release profile of emodin from nanoparticles was presented in **Figure 2D**. In contrast with the emodin solution, there was a significant prolonged time of release of emodin from nanoparticles. For the emodin solution, approximately 100% emodin was released after 8 h, while only 28.69% of emodin was released from the nanoparticles after 72 h. It is indicated that one of the unique characteristics of emodin nanoparticles are to sustain the release of emodin over a long period of time. To further study the release kinetics, the emodin release profile from emodin nanoparticles was analyzed by first-order kinetic model, Higuchi model, and Ritger–Peppas model. The regressed results observed for emodin nanoparticles were *Q* = -0.0029*t* + 4.4413 (*R*^2^ = 0.7531), *Q* = -0.0259*t* + 3.8646 (*R*^2^ = 0.6325), and *Q* = 12.176*t*^0.208^ (*R*^2^ = 0.992), respectively. According to regression coefficient values, a good fit was observed for Peppas equation. As indicated, the value of *n* was <0.5. The result demonstrated that emodin release from the above formulation followed the Fickian diffusion mechanism. Thus, the release profile of emodin can be mainly attributed to the diffusion of the drug from the nanoparticles.

### Biofilm Observation

**FIGURE 3:**
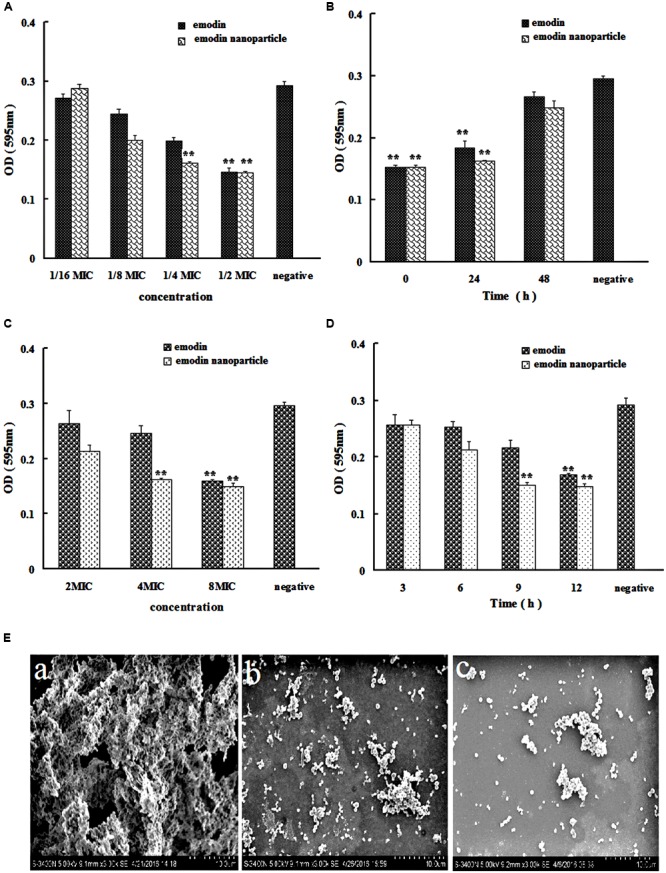
*Streptococcus suis* biofilm formation inhibition and elimination by emodin and emodin nanoparticles. **(A)** *S. suis* biofilm formation inhibition by emodin and emodin nanoparticles with different concentration. **(B)** *S. suis* biofilm formation inhibition by emodin and emodin nanoparticles at different incubation time. **(C)** *S. suis* biofilm formation elimination by emodin and emodin nanoparticles with different concentration. **(D)** *S. suis* biofilm elimination by emodin and emodin nanoparticles at different incubation time. **(E)** SEM of **(a)** *S. suis* biofilm, **(b)** the *S. suis* biofilm incubated with 8× MIC emodin nanoparticles for 12 h, and **(c)** the *S. suis* incubated with 1/2× MIC emodin nanoparticles for 72 h (^∗∗^*p* < 0.05).

The structure of *S. suis* biofilm was observed by SEM. In **Figure 3E(a)**, large number of bacteria were gathered, clumpy structures were formed, and these constitute the main features of biofilm.

### Emodin Nanoparticles Inhibiting Biofilm Formation

The results of MIC analysis experiment indicated that Gel-CD copolymer had no inhibitory effect <4 mg/mL, and the MICs of emodin and emodin nanoparticles against the *S. suis* were determined as 6.25 and 3.12 μg/mL, respectively. MIC of nanoparticles was lower than that of emodin, this could be due to nanoparticles multiple pathways for cellular entry, such as phagocytosis and pinocytosis through clathrin-dependent and clathrin-independent pathways (Sahay et al., 2010).

In the biofilm formation inhibition experiment, TCP assay which determined the formation of bacteria biofilm was used to evaluate the influence of the drugs on biofilm formation *in vitro*. In **Figure 3A**, the emodin nanoparticles could significantly reduce the biofilm formation at 1/4× MIC (0.78 μg/mL) and 1/2× MIC (1.56 μg/mL, *p* < 0.05). And the emodin could inhibit biofilm formation at 1/2× MIC (3.12 μg/mL, *p* < 0.05), showing that the inhibition concentration of emodin was significantly higher than that of the nanoparticles group.

Sub-MIC of emodin (3.12 μg/mL) and 1/2× MIC emodin nanoparticles (1.56 μg/mL) were added to 96-well microplate at different time, respectively, and the wells were tested with TCP assay after incubation for 72 h. **Figure 3B** revealed that both emodin and nanoparticles had significant influence on the biofilm formation after they were incubated for 48 h with the *S. suis*, and the biofilm formation gradually declined with increasing drug incubation time, so the drug inhibition on biofilm is time-dependent.

In **Figure 3E(c)**, the bacteria were dispersedly distributed and the clumpy structures could not be seen, these findings indicated that biofilm was inhibited by 1/2× MIC emodin nanoparticles after incubation for 72 h. This corroborates earlier reports from our laboratory (Zhao et al., 2015), and it is agreed with our previous experimental results.

### Emodin Nanoparticles Eliminating Biofilm

In **Figure 3C**, the biofilm elimination experiment was tested by TCP assay. In comparison with the control group, the 4× MIC emodin nanoparticles (12.5 μg/mL) and 8× MIC emodin (50 μg/mL) significantly reduced the biofilm (*p* < 0.05). In **Figure 3D**, the results showed that the biofilm was destroyed by the drug (which is time-dependent). There was remarkable biofilm destruction after cultivating for 9 h with 8× MIC emodin nanoparticles (25 μg/mL), and the same phenomenon was presented when 8× MIC emodin (50 μg/mL) was incubated with biofilm for 12 h (*p* < 0.05). These data revealed that the emodin nanoparticles group had stronger biofilm elimination ability.

**Figure 3E(b)** revealed that there were little clumpy structures which showed that the bacteria were scattered and the biofilm was eliminated by 8× MIC emodin nanoparticles.

### The Emodin Nanoparticles Uptake of *S. suis* Biofilm and *S. suis*

**FIGURE 4:**
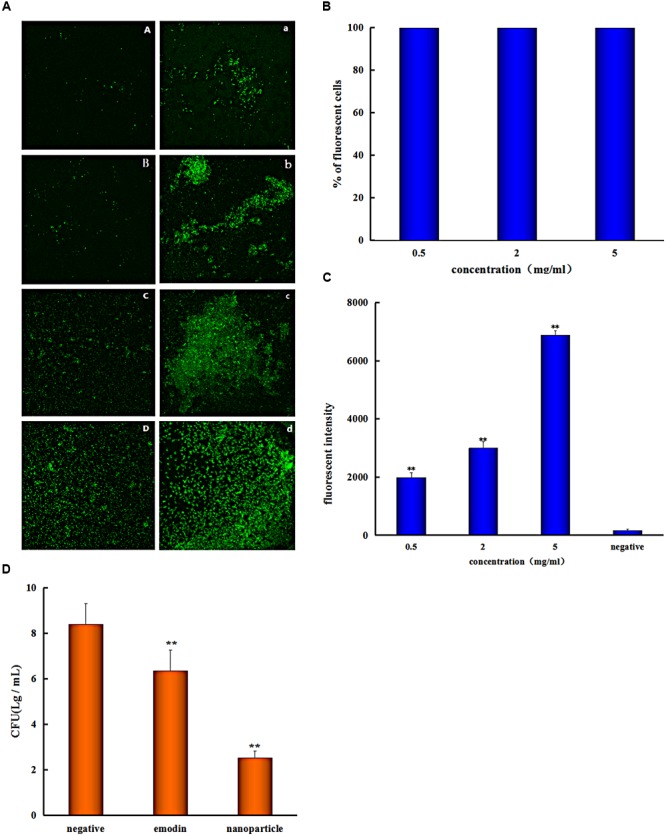
*Streptococcus suis* take in the drug. **(A)** Confocal laser scanning microscopy images of the *S. suis* biofilm treated with coumarin-Sol after 0.25 **(A)**, 0.5 **(B)**, 1 **(C)**, and 3 h **(D)**; the *S. suis* biofilm treated with coumarin-6 nanoparticles after 0.25 **(a)**, 0.5 **(b)**, 1 **(c)**, and 3 h **(d)**. **(B)** Flow Cytometry System measures the percentage of *S. suis* number for coumarin nanoparticles uptake at different concentrations **(C)** and the fluorescent intensity of cells with different coumarin nanoparticles concentrations. **(D)** Colony forming unit (CFU) enumeration of *S. suis* biofilm treaded by emodin and emodin nanoparticles (^∗∗^*p* < 0.05).

The *S. suis* biofilm uptake was investigated by the confocal laser scanning microscopy (CLSM). The *S. suis* biofilm was treated with coumarin-6-labeled nanoparticles for 0.25, 0.5, 1, and 3 h, respectively. There was obvious fluorescence after incubation with biofilm for 0.25 h (**Figure 4A**), this indicated that nanoparticles were able to gather on the biofilm in a short time. And this phenomenon result in the uptake of nanoparticles by bioflim. To investigate uptake capacity of the *S. suis* biofilm, the coumarin-6-labeled nanoparticles were incubated with biofilm for 0.5, 1, and 3 h, respectively, and the fluorescence intensity was gradually increased when drug incubation time was prolonged (**Figure 4A**). The result showed that more coumarin-6-labeled nanoparticles were taken-up by the biofilm with corresponding increase in time.

Biofilm is surrounded by self-produced extracellular polymeric substances (EPSs) which has the ability to reduce the penetration of drug, and the antimicrobial drugs must diffuse through the biofilm matrix in order to inhibit the bacterial cells activity. So EPS represents a strong barrier for these drugs by influencing the rate of its transport into the deep biofilm layer (Kouidhi et al., 2015). Interestingly, it was observed from the results of this study that nanoparticles had better penetration ability for biofilm, and the *S. suis* biofilm uptake increased with corresponding increase in time. Due to the fact that EPS is constituted by proteins, lipids, ions, and nucleic acids, which can form a charged and highly hydrated gel (Sandt et al., 2007), and hydrophilic drugs have better transport ability than their hydrophobic counterparts in biofilm, the drug was covered by Gel-CD copolymer as hydrophilic carrier in the nanoparticles, and thus there was an easy uptake by the *S. suis* biofilm. Furthermore, polysaccharides are major matrix component in most bacterial biofilm (Ganeshnarayan et al., 2009), and the polysaccharides that are positively charged could adsorb nanoparticles by interaction. The phenomenon can also be interpreted by extended Derjaghin–Landau–Verwey–Overbeeck theory (xDLVO), when electrolyte concentration increased outside of bacteria, which may be caused by nanoparticles, the double layer was compressed, the electrostatic interactions, van der Waals interactions, and acid–base interactions between the *S. suis* and nanoparticles were affected, and the adhesion of nanoparticles onto bacteria was also variated (Marshall et al., 1971; Hermansson, 1999; Harimawan et al., 2013). Besides, the size effects on adsorption of nanoparticles on bacteria are reported that the large nanoparticles had better adsorption rate than small nanoparticles when the adsorption rates expressed as mg m^-2^ min^-1^ (Zhang et al., 2010, 2011). So the emodin nanoparticles which were about 174 nm had a better adsorption rate. In **Figure 4A**, nanoparticles easily adhere on the biofilm in a short time, resulting in a better permeation for bacterial biofilm. Improving biofilm uptake can remarkably reduce antibiotic resistance and dramatically improve the therapeutic effects of drugs. This therefore may be one of important reasons for the effective elimination of biofilm by nanoparticles.

Flow cytometry system analysis was performed with nanoparticles and *S. suis* to investigate nanoparticles uptake by bacteria. The result indicated that almost all of the bacteria cells took in nanoparticles at different concentrations (**Figure 4B**), and the fluorescent intensity was enhanced with increased drug concentration (**Figure 4C**). Nanoparticles had better uptake with different concentrations after incubation for 1 h and it is advantageous to antibiosis or antibiofilm property of nanoparticles. Because emodin can inhibit bacterial nucleic acid biosynthesis by disintegrating DNA to small pieces (Huang et al., 2015), and it can also reduce some key proteins expression of ABC transport system, carbohydrate metabolism pathway, and bacterial cell division (Li et al., 2017) when there is uptake of the drug. And the results are consistent with MIC analysis experiment and biofilm formation inhibition.

### Colony Forming Unit (CFU) Enumeration

To better assess the penetration ability of emodin nanoparticles on biofilm, the CFUs of *S. suis* were counted. The viability of *S. suis* biofilm treated with 25 μg/mL emodin or emodin nanoparticles was different from the viability of untreated *S. suis*. The number of CFUs/mL in emodin-treated biofilm (6.3 log10 CFUs/mL) was fewer than those in nontreated biofilm (8.4 log10 CFUs/mL; *p* < 0.05) and the CFUs/mL number of emodin nanoparticles treated biofilm (2.5 log10 CFUs/mL) was significantly less than emodin biofilm (6.3 log10 CFUs/mL) (**Figure 4D**). The findings demonstrated that there was an easier and faster uptake of emodin nanoparticles by biofilm, and more *S. suis* was eliminated when it took in more of the drug.

## Conclusion

A novel type of self-assembled Gel-CD copolymer was synthesized. And the emodin was successfully incorporated into nanoparticles to form shuttle-shape emodin nanoparticles. The nanoparticles had many excellent features including their zeta potential, size distribution, DL, and sustained-release profile. In addition, emodin nanoparticles enhanced the inhibition of biofilm formation and biofilm elimination *in vitro*, and it presented a better condition for drug uptake by biofilm or bacteria.

## Author Contributions

YhL and JS designed the experiments. WD participated in all the experiments and wrote the manuscript. BG, XC and YyL modified the manuscript. HL, DZ and XH took part in the preparation of emodin nanoparticles. CX, XL, and DZ acted the experiments of nanoparticles delivery situation. SZ took part in the experiments of colony forming unit enumeration.

## Conflict of Interest Statement

The authors declare that the research was conducted in the absence of any commercial or financial relationships that could be construed as a potential conflict of interest.
